# Depressive Symptoms Mediate the Influence of Fibromyalgia Status on Physical Performance and BMI in Aging Adults

**DOI:** 10.1093/geroni/igab046.3108

**Published:** 2021-12-17

**Authors:** Dylan Serpas, Laura Zettel-Watson, Barbara Cherry

**Affiliations:** 1 California State University, Fullerton, Bonita, California, United States; 2 California State University, Fullerton, Fullerton, California, United States

## Abstract

Fibromyalgia is a chronic pain condition that is frequently accompanied by comorbid conditions, including depression. Depression is associated with reduced physical functioning and health disproportionately affecting middle-aged and older adults with fibromyalgia. This study examined depressive symptoms as a mechanism through which FM status is associated with BMI and physical performance among adults in mid-to-late-life. Participants included 250 community-dwelling middle-aged and older adults (82% female) with (59%) or without (41%) fibromyalgia (M age = 64.44, SD = 9.16). Depressive symptoms were measured using the Beck Depression Inventory-II, BMI was objectively assessed, and physical performance was measured using the Fullerton Advanced Balance scale, 6-Minute Walk Test, 30-Second Chair Stand, and 8-Foot Up and Go Test. Physical performance measure analyses were adjusted for age. Asymptotic mediation analyses revealed that fibromyalgia status was indirectly associated with higher BMI (95% CI [.18, 16.74]), and poorer performance in the Fullerton Advanced Balance (CI [-2.93, -1.24]), 6-Minute Walk Test (CI [-73.75, -35.35]), 30-Second Chair Stand (CI [-2.45, -1.16]), and 8-Foot Up and Go test (CI [.35, .92]) via depressive symptoms. Participants with fibromyalgia reported greater depressive symptoms which was subsequently associated with greater BMI and reduced physical performance. Findings support depressive symptoms as one factor through which fibromyalgia status is associated with higher obesity risk and reduced physical function in middle-aged and older adults with fibromyalgia. This study supports fibromyalgia status as a critical consideration when evaluating the health and disability risk of aging adults.

